# Case of Stricture of the Bowels

**Published:** 1851-10

**Authors:** W. A. Smith

**Affiliations:** Rutherford Co., Ten.


*Case of Stricture of the Bowels.* By W. A. Smith, M. D., of Rutherford Co., Ten.—The history of the case about to be submitted is as follows:—J. W., aged 43 years, had chronic rheumatism for fifteen or twenty years previous to his death, and it became so troublesome that he had to abandon his profession; had been addicted to the excessive use of alcoholic stimulants for fifteen or sixteen years; sometime previous to his last illness, he had been troubled with dyspeptic symptoms, for the relief of which he had visited the mineral wells in West Tennessee; during his stay in the District, had a slight diarrhœa, and health generally improved. A short time after his return to this county he was attacked in August last, with great pain in his bowels, with manifest fullness in the direction of the colon, and considerable borborygmus. Dr. Black was called in, to whom the patient stated, that for several months he had frequent attacks of colic, and that his bowels were very costive—rarely being moved, except from the use of purgatives.

This pain in the bowels came on in paroxysms, at intervals of fifteen or twenty minutes, and lasted about thirty seconds. During the paroxysms he suffered excruciating pain, and great sensibility of the walls of the abdomen—so much so that he could not bear the slightest pressure without increase of pain, and upon the subsidence of the paroxysm no pain was elicited by pressure. He stated that the above had been his condition for several days—and that he had gradually grown worse; that heretofore he had relieved himself with a pill composed of rhubarb and aloes; this remedy had now been tried, and failed to open his bowels. He had used a number of the domestic remedies for colic before the Doctor arrived, such as peppermint, camphor, and paregoric, but all to no effect. He now took a full dose of oleum ricini, with half an ounce of paregoric, and had flannel cloths wrung out of warm water applied to his abdomen—there being no arterial excitement indicating bloodletting.

After waiting some time, and no motion from his bowels, a stimulating enema was administered, which, according to the Doctor's statement, brought off a small discharge of indurated feces and some gas.

Partial freedom from pain was thus obtained, when the patient was left, with orders to take twenty grains of calomel, one grain of ipecac, and half a grain of sulphate morphia, to be taken in three hours, if his bowels were not moved again; to be followed (in case his bowels should not be moved within the usual time) with a full dose of oleum ricini, and a teaspoonful of turpentine; fomentations continued, and enema to be repeated, if necessary.

Visited by Dr. Black on the next day; medicine had acted very imperfectly, and patient but little if any better. A turpentine enema was now given, which procured a free evacuation, followed by relief of the pain for a time at least. Prescribed a powder composed of five grains calomel, half grain ipecac, to be repeated every four hours until four in number had been taken, and if purging was not produced, to be followed by oil and turpentine, and the enema, as before, if necessary.

Visited the next day; remedies had all been used, and only moderate purging obtained, though the patient appeared to be much better, and thought he would be up soon. He was ordered to take some pills composed of blue mass, rhubarb, com. ext. of colocynth, so as to keep up the action of the bowels.

Nothing heard from him for four days, when Dr. Black was sent for again, and found the patient laboring under all the symptoms previously described—only with more violence. He was ordered to take croton oil (say two drops) in an ordinary dose of castor oil, with directions to repeat the dose at intervals of four hours, if necessary. The dose was repeated, and the pain growing worse, the attending physician was called in again.

At this visit the patient is represented to be suffering the most excruciating pain, coming on at intervals and lasting longer than they had previously done—the subsidence of the paroxysm was attended with a gurgling, like unto water escaping from a jug. His abdomen is now quite tympanitic, and very tender on pressure—more particularly when the paroxysm is on. This gurgling sound within the bowels was universally attended with a mitigation of all the urgent symptoms—it at all times appeared to take the direction of the colon—sometimes the sound began in the left, and at other times in the right side.

The sufferings of the patient were now greatly increased by the violent efforts to expel the contents of the bowels, which was prevented by some obstruction as was supposed—either spasm, intussusception, stricture, or indurated feces.

With this view of the case, purgatives were discontinued, and free leeching and fomentations were used, with as much of the acetate morphia as might be necessary to quiet the action of the bowels and secure an exemption from pain.

About this time, Dr. Jas. E. Wendel and Dr. Avent saw the case, and it was agreed that a flexible bougie, twenty-two inches in length, should be passed up the rectum in search of some obstruction. The withdrawal of the instrument was followed by an evacuation of feculent matter, which brought but little relief to the patient. Leeching, fomentations, blisters, alternate doses of calomel, and the internal use of anodynes, constituted the treatment for several days, with little or no variation in the symptoms.

About the 4th or 5th of September, five of the faculty called to see the case, and just before their arrival the patient had a copious fluid evacuation, passing tomatoe seed, which had been eaten a week previously. All the symptoms appeared to be greatly relieved for several days. The warm bath, alternate doses of mercury, combined with hyosciamus, stramonium, cicuta, morphia and ipecac, and solutions of the iodide of potash, constituted the treatment for several weeks, with but little variation in the symptoms. From the first of September, the patient was kept almost continually under the influence of narcotics, and whenever these were stopped, his pain would return with great severity. For three months previous to his death, he rarely had a motion from his bowels only when under the influence of opiates. During his entire sickness, his constitution was never reached by the mercurials, though they had been employed freely, both externally and internally.

About the 1st of November, Dr. B. took a trip to the South, but before leaving requested me to see his patient occasionally. I did so. At this time the patient was recovering from an unusually free attack of watery purging. He continued to take the morphia, and, in fact, there appeared to be no freedom from pain only when under its influence. As almost every thing had been tried, I determined to put him on quinine and the precipitated carb. iron, with the view of meeting the paroxysmal indications. This was kept up for two or three weeks, but brought no relief. The quinine and iron were then withheld, and the tinct. of poke root tried to no effect. About the 25th of November, he complained of some fullness in his bowels, which was soon followed by swelling of the feet. At the suggestion of Dr. Rucker, the tinc. of the pokeberry was given in place of that made from the root, but no benefit was derived from the change, the patiently gradually sinking at the time.

On Christmas morning, I called to see him; found Dr. Black there; learned that the patient had sat up that morning, and expressed himself better than usual, and all at once, without any assignable cause, went into a profound coma, out of which he was never aroused. When he was in articulo mortis, his pulse was remarkably good, say numbering about 90 and regular, with volume enough to indicate that blood was thrown to the extremities. On the next day, which was the 26th of September, Dr. B. and myself made a post mortem examination; found about three pints of serum in the peritoneal cavity; the whole serious membrane lining the cavity of the abdomen, thickly studied with miliary tubercle; several points of illium highly inflamed, and of recent origin; the entire colon was very much thickened and indurated, and enlarged, except two strictured points; cecum also inflamed; one of the contracted points of the colon was just above where it unites with the rectum, and the other near caput coli. The contractions were about two inches in length, and from one-quarter to one-half inch in diameter, and when cut into, presented the appearance of a fibro-cartilaginous substance. The stomach was very much contracted, with its wall thickened to a considerable degree, in fact, the whole alimentary canal was literally a mass of disease.

In connection with this report, imperfect as it must necessarily be, permit me to submit to the inspection of the Society some nondescripts, (if I may use the expression,) obtaned from a section of the colon.—*Ibid.*

